# Hard carbon anodes for sodium-ion batteries: from mechanistic reconciliation, precursor-tailored microstructure regulation to industrial scale-up

**DOI:** 10.3389/fchem.2026.1978945

**Published:** 2026-10-29

**Authors:** Yong-Gang Sun, Jin-Yi Ding, Jian Xiong, Yi-Han Zhang, Hao-Jie Fan, Yi-Cong Xie, Qi-Rui Zhang, Kang-Lin Zhang, Feng-Cai Li, Bei-Bei Zhang

**Affiliations:** School of Chemistry and Chemical Engineering, Yancheng Institute of Technology, Yancheng, China

**Keywords:** commercialization, hard carbon, precursor, sodium-ion battery, structural model

## Abstract

Hard carbon is the leading anode candidate for sodium-ion batteries, yet its practical deployment remains constrained by three interconnected gaps: the sodium storage mechanism is disputed across competing models, no precursor classification framework links chemical composition to electrochemical function, and predictive structure–property relationships remain largely empirical. This review traces the precursor-to-performance pipeline. We show that the insertion-filling, adsorption-insertion, and adsorption-filling models converge on a multi-stage adsorption-intercalation-filling paradigm, where the intercalation-to-filling balance is governed by carbonization temperature, precursor chemistry, and pore architecture rather than by a single mechanism. We then introduce a three-dimensional precursor classification framework—spanning scalability, structural controllability, and performance ceiling—and deconvolute the component-specific pyrolysis pathways of cellulose, hemicellulose, and lignin to establish how molecular architecture is transmitted into carbon microstructure. Three microstructure engineering levers are critically evaluated: pore-structure manipulation to resolve the low-surface-area/high-closed-pore-volume trade-off, interlayer-spacing regulation via heteroatom doping and pre-oxidation, and defect engineering guided by the conductivity-capacity Pareto Frontier. The rapid industrialization of hard carbon—from laboratory discovery to 10,000-ton-scale manufacturing—is assessed, documenting how precursor diversification and process innovation have driven costs toward parity with graphite anodes. Finally, the current challenges and future development directions of high-performance and industrializable hard carbon anodes are prospected, aiming to provide systematic theoretical guidance and technical references for the further design, modification and large-scale application of hard carbon materials in advanced sodium-ion batteries.

## 1 Introduction

Grid-scale electrochemical energy storage is central to decarbonization (Yao et al., 2025). Lithium-ion batteries (LIBs), despite their technological maturity, face mounting sustainability pressures from the limited crustal abundance of lithium (0.002%) and geographically concentrated supply chains (Greim et al., 2020) of cobalt and nickel. Sodium-ion batteries (SIBs) have emerged as the most promising near-term alternative, leveraging sodium’s natural abundance (Yao et al., 2025) (2.36% of the Earth’s crust), the structural similarity to LIBs in operating principles and manufacturing infrastructure, and the economic advantage of aluminum current collectors on both electrodes. However, graphite—the dominant commercial LIB anode—delivers negligible sodium storage capacity due to thermodynamically unfavorable Na^+^-graphite intercalation, necessitating a fundamentally different anode paradigm (Tang et al., 2023). Hard carbon (HC) stands out as the most industrially viable choice, owing to its disordered turbostratic structure with expanded interlayer spacing (d_002_ = 0.36–0.41 nm), abundant nanoporosity, and excellent cycling stability (Tang et al., 2023; Wu C. et al., 2025).

Hard carbon is a non-graphitizable carbon whose microstructure comprises three-to-seven-layer curved graphene sheets assembled into short-range ordered pseudo-graphitic domains, interspersed with nanoscale pores (open, closed, and sieving) and structural defects of both intrinsic (vacancies, edges) and extrinsic (heteroatom dopant) origin (Sun et al., 2025; Chu et al., 2023; Liu et al., 2024; Su et al., 2024). This multi-scale heterogeneity endows HC with a broad sodium storage capability spanning pseudocapacitive adsorption at defects and pore surfaces (the sloping region above 0.1 V vs. Na/Na^+^), intercalation between pseudo-graphitic interlayers, and quasi-metallic sodium cluster filling within closed nanopores (the plateau region below 0.1 V) (Lu et al., 2024). Electron paramagnetic resonance spectroscopy recently corroborated this multi-mechanism picture by quantitatively distinguishing Faradaic intercalation from capacitive adsorption during cycling (Wang B. et al., 2024). Since Stevens and Dahn’s introduction of the House of Cards model in 2000, the field has accumulated substantial experimental and computational evidence, yet three central challenges remain unresolved (Sun et al., 2025; Ste et al., 2000).

The sodium storage mechanism remains debated (Zhang et al., 2025a). Three competing models (Wu et al., 2024) insertion-filling (sloping = intercalation, plateau = pore filling), adsorption-insertion (sloping = adsorption, plateau = intercalation), and adsorption-filling (sloping = adsorption, plateau = pore filling)—each assigns the sloping and plateau capacities to different structural sites, and the discrepancies between these interpretations have hindered the formation of a unified consensus framework. Beyond mechanistic uncertainties, although biomass represents the most sustainable and scalable HC precursor class (Huang et al., 2024), the field lacks a classification system that simultaneously accounts for chemical composition, industrial scalability, and achievable structural control. Recent operando studies demonstrate that even within a single biomass feedstock, cellulose (Xie et al., 2022) and lignin follow divergent pyrolysis pathways that produce fundamentally different pore architectures, with cellulose favoring open microporous networks and lignin promoting closed-pore (Zhang et al., 2024) formation through its intrinsically crosslinked aromatic structure. Compounding these knowledge gaps, the quantitative mapping from precursor chemistry through pyrolysis pathways to carbon microstructure and ultimately sodium storage performance remains largely empirical, with data-driven approaches only beginning to bridge this gap (Jiang et al., 2025).

This review reconciles the three competing sodium storage models—insertion-filling, adsorption-insertion, and adsorption-filling—within a unified multi-stage adsorption-intercalation-filling paradigm, revealing that their apparent contradictions arise from the sensitivity of the intercalation-to-filling balance to carbonization temperature, precursor chemistry, and pore architecture (Lu et al., 2024; Li et al., 2024; Ouyang et al., 2026). The four key governing parameters—carbonization temperature, heteroatom doping, pore structure, and precursor selection—are then examined in depth, with particular attention to the transition from open micropore-dominated surface adsorption to closed-nanopore cluster filling as the dominant low-potential plateau storage channel. A three-dimensional precursor classification framework encompassing biomass, sugars, resins, and pitch is subsequently established, within which the component-specific pyrolysis pathways of cellulose, hemicellulose, and lignin are deconvoluted to trace how their molecular architecture is transmitted into the final carbon microstructure (Joseph et al., 2025). Finally, the rapid industrialization trajectory of hard carbon is assessed, from laboratory discovery to 10,000-ton-scale manufacturing, analyzing the market dynamics, cost-reduction pathways, and supply-chain restructuring that have established China as the dominant global production hub, with hard carbon approaching cost parity with lithium-ion battery graphite anodes (Figure 1).

**FIGURE 1:**
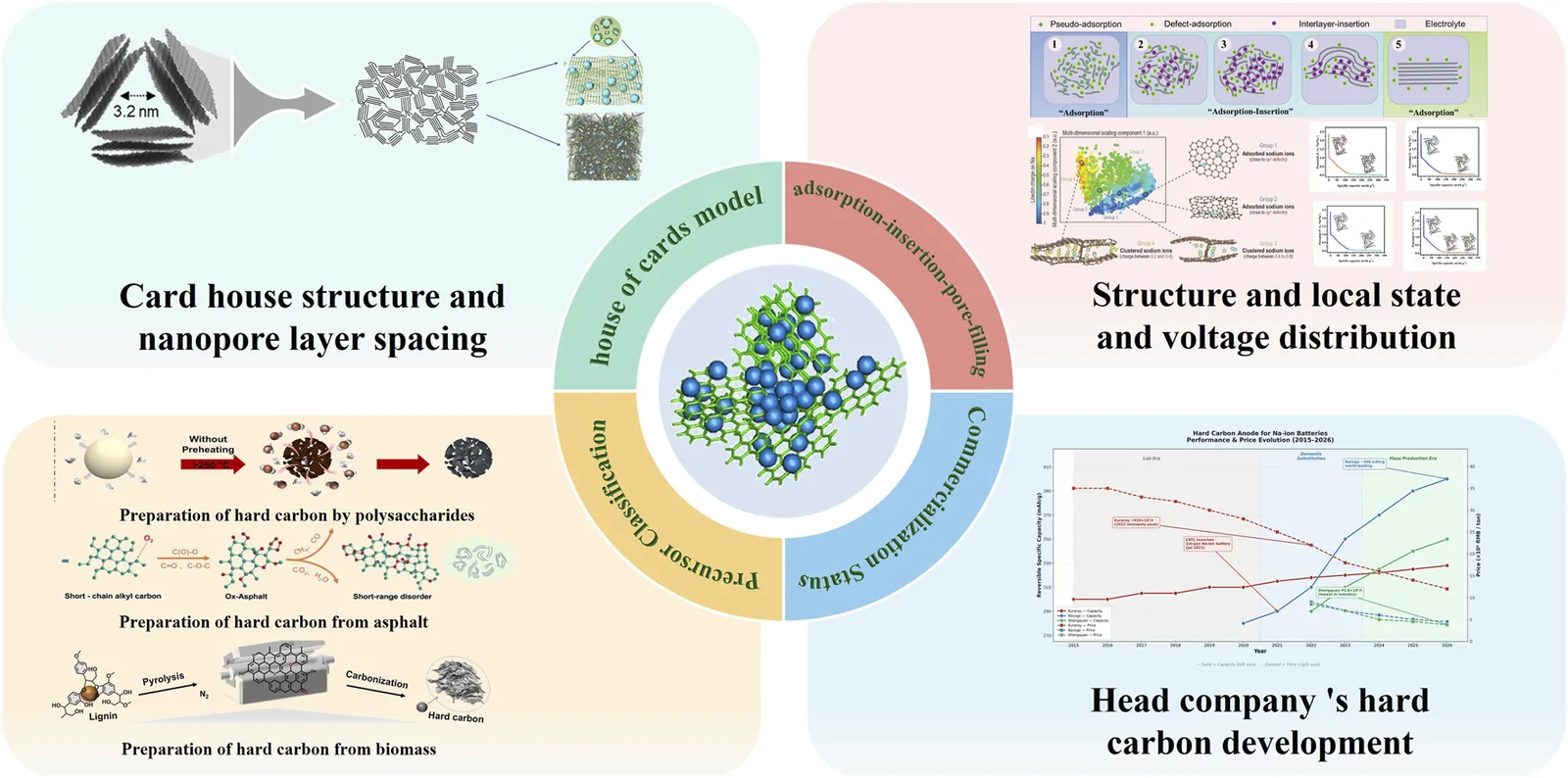
Hard carbon structure mechanism and commercial distribution strategy diagram.

## 2 Hard carbon microstructure and sodium storage mechanisms

### 2.1 Hard carbon microstructure model

Hard carbon belongs to the class of non-graphitizable carbons that retain their disordered turbostratic structure even upon heating above 3000 °C. In 1997, Dahn et al. first proposed the “falling cards” (or “House of Cards”) (Figure 2a) model, describing HC as an assembly of small aromatic fragments approximately 40 Å in lateral dimensions that are randomly oriented and stacked—much like scattered playing cards—which naturally gives rise to two types of structural sites: locally parallel graphene sheets of 2–3 layers providing interlayer insertion sites, and nanoscale voids between randomly oriented layers constituting micropores (Ste et al., 2000). In 2000, Stevens and Dahn extended this model to the sodium system. By comparing the electrochemical behavior of glucose-derived HC (pyrolyzed at 1000 °C and 1150 °C) in Li and Na half-cells, they demonstrated that both exhibit the same two-region profile: the sloping region originates from ion intercalation between quasi-parallel layers (the turbostratic stacking produces a continuous distribution of site potentials, resulting in gradually decaying voltage with capacity), while the low-potential plateau region is attributed to adsorption-like filling of ions in nanopores, where the chemical potential of the inserted species approaches that of the bulk metal (Dahn et al., 1997). They further demonstrated that the nanoporosity estimated from Na and Li storage was approximately 20 vol% and 21 vol%, respectively, and the ratio of plateau capacities (42:77 for Na:Li) matched the molar volume ratio of the two metals, providing quantitative support for the House of Cards model. Recently, Tang explored the structural model of hard carbon storage sodium ions by developing a new type of material to explore the hard carbon bicontinuous structure, and found that the existence of 3D continuous channels and frameworks facilitated rapid and uniform transport of Na ions, improving the transport dynamics of the HC anode (Figures 2b–e). Three distinct sodium storage processes were identified within precise voltage ranges for the SP–HC anode: surface adsorption, intercalation, and nanopore filling (Tang et al., 2026).

**FIGURE 2:**
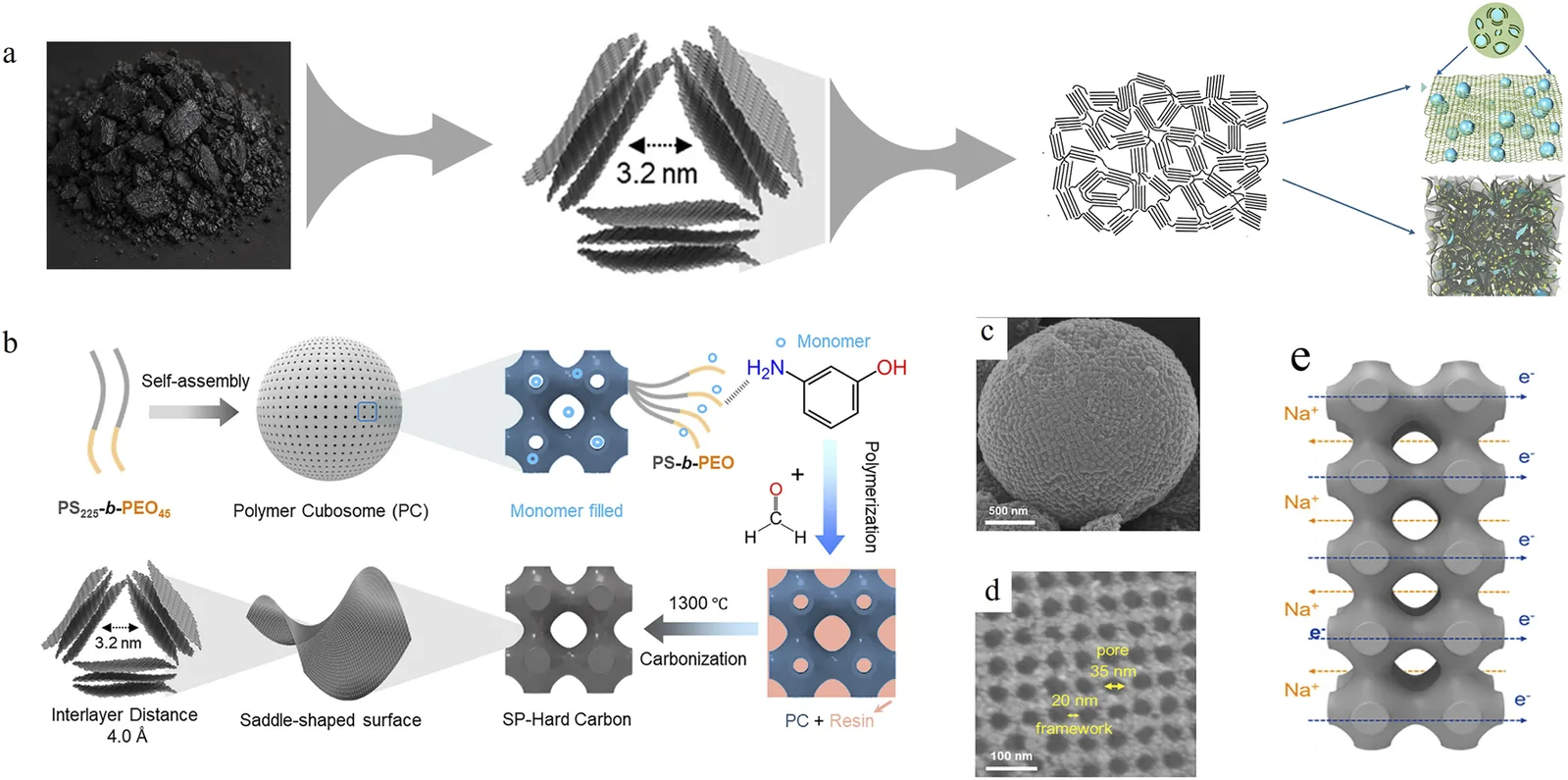
**(a)** Schematic diagram of graphite microstructure (Ste et al., 2000), **(b)** Schematic preparation of hard carbon cubosomes with an SP bicontinuous structure, **(c)** SEM image of an SP–HC cubosome, **(d)** SEM image of a fractured internal section of SP–HC, the average mesopore diameter is 35 nm, and the average frame diameter is 20 nm, **(e)** chematic diagram of a 3D ordered transport mode of Na and electrons in SP–HC with a bicontinuous structure (Tang et al., 2026).

### 2.2 Three models of sodium storage mechanism

The charge-discharge profile of HC typically exhibits two voltage regions: a high-potential sloping region (>0.1 V vs. Na/Na^+^) and a low-potential plateau region (<0.1 V). The structural assignment of the capacity in each region has been the subject of extensive debate, with three competing models proposed successively (Figure 3c). The essence of their disagreement lies in the structural origin of the plateau capacity. By analyzing the relationship between microstructure and sodium storage performance, Sun et al. (2019) proposed an extended ‘adsorption-embedding ‘model based on theoretical calculations (Figure 3a). Li studied the scalable low-voltage platform technology of sieved hard carbon and proposed the sodium storage mechanism. Na^+^ in the slope area preferentially adsorbs on the surface of hard carbon microspheres, and the platform area mainly includes Na^+^ insertion between graphite-like microcrystalline layers (Fan et al., 2026). In addition, a simplified model of sieved carbon was built based on machine learning to study the preferential enrichment position of sodium clusters in the platform area (Figure 3b).

**FIGURE 3:**
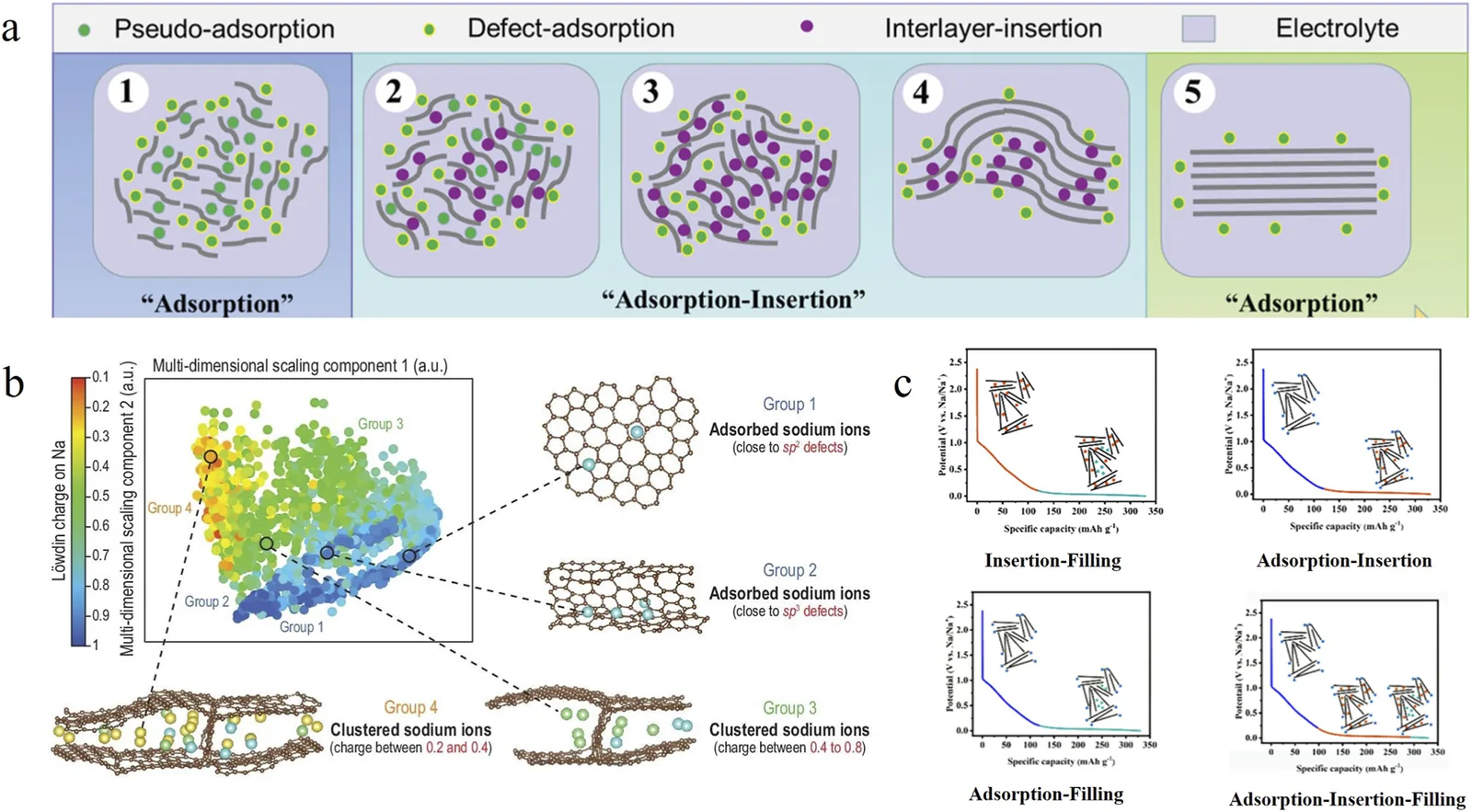
**(a)** The evolution of the microstructure (Sun et al., 2019), **(b)** Localized state of sodium atoms in screened carbon (Li et al., 2022), **(c)** The voltage range and extension diagram of the three models.

#### 2.2.1 Insertion-filling model

The insertion-filling model, originating from the pioneering work of Stevens and Dahn on glucose-derived HC, assigns the sloping region to Na^+^ intercalation into graphitic interlayers and the plateau region to Na^+^ filling within nanopores. In the 2020s, this model underwent systematic revision. Alptekin et al., employing ^23^Na ssNMR, PDF, and SAXS on a systematically tuned series of HC samples, discovered that Na^+^ intercalation and pore filling are not simply sequential: within the sloping region, Na^+^ simultaneously and continuously stores at defect sites, interlayers, and pore surfaces—three processes coexisting rather than a single mechanism dominating—and only after these higher-energy binding sites are occupied does closed-pore filling occur during the plateau region, with the confined sodium taking on increasingly metallic character (Au et al., 2020). In their 2024 continuation work, Dahn’s group further developed this revision into a “wedge nanopore” model: during the initial discharge, Na^+^ first adsorbs on edge and vacancy defects (sloping region), subsequently enters the narrow end of closed pores where the confinement effect induces Na^+^ desolvation and the formation of 1–2 quasi-metallic Na layers covering the pore-wall carbon surface, analogous to underpotential deposition (UPD) on metal electrodes; DFT calculations indicate an optimal pore size of approximately 1 nm accommodating 2–3 Na layers (Li et al., 2024). This work explains the fast-charging capability of HC from a kinetic dimension—the reduced desolvation barrier in narrow pore channels is the origin of fast charging—thereby unifying thermodynamic assignment and kinetic behavior within the insertion-filling framework.

#### 2.2.2 Adsorption-insertion model

The adsorption-insertion model assigns the two voltage regions inversely: the sloping region originates from pseudocapacitive adsorption of Na^+^ on defects and surfaces, while the plateau region originates from Na^+^ intercalation into graphitic interlayers. In 2021, Han et al. prepared a series of hard carbons ranging from completely disordered to progressively ordered through low-temperature dealloying, establishing a quantitative correlation between local atomic structure and electrochemical behavior via PDF and molecular dynamics simulations. They not only confirmed the core tenet of the adsorption-insertion model—that low-potential plateau capacity increases with graphitic ordering—but also identified a previously unrecognized medium-potential sloping adsorption mechanism: Na^+^ adsorption on defective sites of graphitic domains (such as vacancies and edges) contributes additional capacity between fully disordered carbon adsorption and interlayer intercalation, thereby refining the traditional two-stage model into a three-stage picture: disordered carbon adsorption (>0.4 V), graphitic defect adsorption (0.1–0.4 V), and interlayer intercalation (<0.1 V) (Han et al., 2021). Ouyang and Zhong et al. further captured the site occupancy transition at the atomic/electronic level using operando ^23^Na ssNMR and ^13^C NMR: during the initial discharge, the signal at ∼−30 ppm (interlayer NaC_x_) and ∼−15 ppm (defect-associated NaC_a_) grow simultaneously; upon entering the plateau region, both begin to decay—with interlayer sodium content approaching near-zero in low-defect HC—while the quasi-metallic sodium cluster signal (∼884–1024 ppm) accelerates in growth during the plateau region (Ouyang et al., 2026). This dynamic site migration from intercalated and defect-bound Na toward closed-pore Na clusters—rather than simple sequential filling—provides the first direct spectroscopic evidence that the primary plateau capacity originates from closed-pore Na clusters rather than sustained intercalation, and that the carbon layers themselves (as pore walls) undergo responsive electronic-state changes during the process.

#### 2.2.3 Adsorption-filling model

The adsorption-filling model maintains that the sloping region arises from defect/surface adsorption while the plateau region is exclusively attributable to Na^+^ filling/clustering within nanopores, denying any substantive contribution from interlayer intercalation to the plateau capacity. This model received its strongest theoretical support from DFT calculations by Youn et al., who systematically evaluated the thermodynamic stability of three sodium storage modes in HC: single-atom adsorption on defective graphene is exothermic (≈−1 eV), serving as the source of sloping capacity; 3–6 layer Na nanoclusters—rather than single atoms or complete metallic plating—are energetically favored in model micropores between two graphene sheets, with their calculated potential corresponding to the plateau region (<0.1 V); critically, the DFT-determined capacity limit of the intercalation mechanism is only approximately 140 mAh g^−1^, meaning the deficit to >300 mAh g^−1^ capacity in typical HC must be compensated by pore filling, with the 478 mAh g^−1^ capacity of MgO-templated HC corresponding to enhanced cluster filling in appropriately sized micropores (Youn et al., 2021).

Chen and Cao et al. further bridged the gap between the three models through experimental investigation (Chen et al., 2022). They prepared two structurally distinct HCs: Glu (rich in pseudo-graphitic nanodomains, closed-pore volume 0.084 cm^3^ g^−1^) and Mg-Glu (rich in open micropores, closed-pore volume only 0.002 cm^3^ g^−1^). Through combined operando XRD, *ex situ* Raman, ssNMR, and DFT calculations, they found that the plateau capacity sources were fundamentally different between the two carbons—Glu’s plateau capacity was dominated by interlayer intercalation (interlayer Na signal prominent in ssNMR), while Mg-Glu’s plateau capacity was dominated by micropore filling (quasi-metallic Na cluster signal dominant)—and accordingly proposed an adsorption-intercalation/filling hybrid mechanism: the sloping region is uniformly contributed by defect/surface adsorption, while the plateau region accommodates simultaneous intercalation and pore filling, with their relative weighting determined by the carbon’s microstructural configuration—specifically the balance between pseudo-graphitic domain development and closed-pore volume.

Synthesizing the trajectory of these models since 2020, a clear pattern emerges. First, the structural assignment of the sloping region has largely reached consensus: defect site adsorption (including heteroatoms, vacancies, and edges) and carbon surface physical adsorption jointly contribute the sloping capacity, and the earlier proposition of “sloping = intercalation” is no longer supported by mainstream experimental evidence. Second, the source of plateau capacity—interlayer intercalation versus closed-pore filling—remains the central point of contention, but the debate paradigm has shifted from “mutually exclusive binary choice” in the 2010s to “coexistence with different weighting” in the 2020s. Alptekin’s revised insertion-filling model (Au et al., 2020), Chen and Cao’s hybrid mechanism (Chen et al., 2022), and Ouyang and Zhong’s site occupancy transition (Ouyang et al., 2026) collectively indicate that: after the sloping region, Na^+^ first intercalates into pseudo-graphitic interlayers (contributing transition-region capacity at 0.05–0.1 V), following which—as intercalation sites approach saturation—quasi-metallic sodium cluster filling within closed pores becomes the dominant storage channel (contributing the primary plateau capacity below 0.05 V). The relative contributions of intercalation and filling are governed not by a single variable but by the three-dimensional parameter space of pseudo-graphitic domain interlayer spacing (d_002_), closed-pore volume, and pore-wall defect density—all shaped by carbonization temperature, precursor molecular structure, and template strategy (Ouyang et al., 2026; Youn et al., 2021). From DFT calculations (Youn et al., 2021) to operando spectroscopy (Ouyang et al., 2026; Au et al., 2020) to quantitative structure-performance correlations (Han et al., 2021; Chen et al., 2022), these three lines of evidence mutually reinforce and progressively converge, moving a field once divided into three mutually exclusive positions toward a unified multi-stage, multi-active-site synergistic framework. A comparison of three sodium storage mechanism models for hard carbon is shown in Table 1.

**TABLE 1:** Comparison of the three sodium storage mechanism models for hard carbon.

Model (origin)	Sloping region (>0.1 V) assignment	Plateau region (<0.1 V) assignment	Main active sites	Key characterization techniques	Limitations
Insertion–Filling Stevens and Dahn; revised by Alptekin et al. Au et al. (2020) and Dahn’s “wedge nanopore” model (Li et al., 2024)	Na^+^ intercalation into pseudo-graphitic interlayers (revised: Coexisting storage at defects, interlayers, and pore surfaces)	Na^+^ filling in nanopores/closed pores; confined Na becomes increasingly quasi-metallic	Graphitic interlayers; nanopores/closed pores; (revised) defect sites and pore-wall surfaces	Na ssNMR, PDF, SAXS; DFT (optimal pore ≈1 nm, 2–3 Na layers)	“Sloping = intercalation” premise overturned; sequential two-stage picture oversimplified (three processes coexist)
Adsorption–Insertion Han et al. (2021) refined by Ouyang and Zhong	Pseudocapacitive adsorption on defects/surfaces; refined three-stage: Disordered-carbon adsorption (>0.4 V) and graphitic-defect adsorption (0.1–0.4 V)	Na^+^ intercalation into graphitic interlayers	Defect/surface sites; graphitic defects (vacancies, edges); graphitic interlayers	PDF + molecular dynamics operando^23^Na ssNMR &^13^C NMR (interlayer NaCx ≈−30 ppm; defect NaCa ≈−15 ppm; quasi-metallic Na ≈884–1024 ppm)	“Plateau = intercalation” challenged — plateau capacity mainly from closed-pore Na clusters, not sustained intercalation (low-defect HC)
Adsorption–Filling DFT Youn et al. (2021); Chen and Cao et al. (Chen et al., 2022)	Defect/surface adsorption	Exclusively Na^+^ filling/clustering in nanopores (no intercalation contribution)	Defect/surface sites; nanopores/micropores (3–6 layer Na nanoclusters)	DFT (intercalation capacity limit ≈140 mAh g^−1^) operando XRD, *ex situ* Raman, ssNMR	“Filling-only” too absolute — pseudo-graphitic carbons (Glu) show interlayer-intercalation-dominated plateau

### 2.3 Key factors governing sodium storage

The foregoing discussion of these models reveals that the relative weighting of intercalation versus pore filling is not dictated by a single factor but by the unique microstructural parameter space of each carbon material. Four core structural factors—carbonization temperature, precursor chemistry, heteroatom doping, and pore architecture—act in concert to shape the dominant sodium storage behavior exhibited by a given carbon material.

#### 2.3.1 Carbonization temperature

Carbonization temperature is the primary thermodynamic parameter governing hard carbon microstructure, simultaneously directing three coupled processes: the progressive elimination of structural defects (sp^3^ carbon and heteroatom functional groups), the lateral growth and stacking of pseudo-graphitic domains, and the evolution of pore architecture from open micropores toward closed nanopores. At low temperatures (800 °C–1200 °C), the carbon skeleton retains abundant oxygen-containing functional groups, high defect density, and large interlayer spacing (d_002_ > 0.38 nm), with sodium storage dominated by pseudocapacitive adsorption at defect sites and heteroatom-functionalized surfaces, producing a pronounced sloping voltage profile but low initial Coulombic efficiency (Kim et al., 2025; Qin et al., 2026). Among them, Liang et al. (Iglesias et al., 2023) systematically used XRD to display crystal structures at different temperatures (Figure 4). As the temperature rises into the intermediate window (1200 °C–1600 °C), residual sp^3^ carbon is progressively converted to sp^2^ graphitic carbon, oxygen crosslinking sites are largely eliminated, and carbon-layer curvature-driven rearrangement (Zhou Z. et al., 2026) closes open micropores into discrete closed nanopores; this regime maximizes closed-pore volume while retaining sufficient interlayer spacing (d_002_ = 0.37–0.39 nm) for Na^+^ intercalation, and the plateau capacity below 0.1 V increases sharply as closed-pore filling becomes the dominant storage channel (Deng et al., 2026; Kuang et al., 2026). At excessively high temperatures (>1700 °C), intensified graphitization narrows d_002_ below 0.37 nm and eliminates most closed porosity, impeding both intercalation and pore filling, which results in a catastrophic decline in total capacity (Li R. et al., 2026; Huang Z. et al., 2025).

**FIGURE 4:**
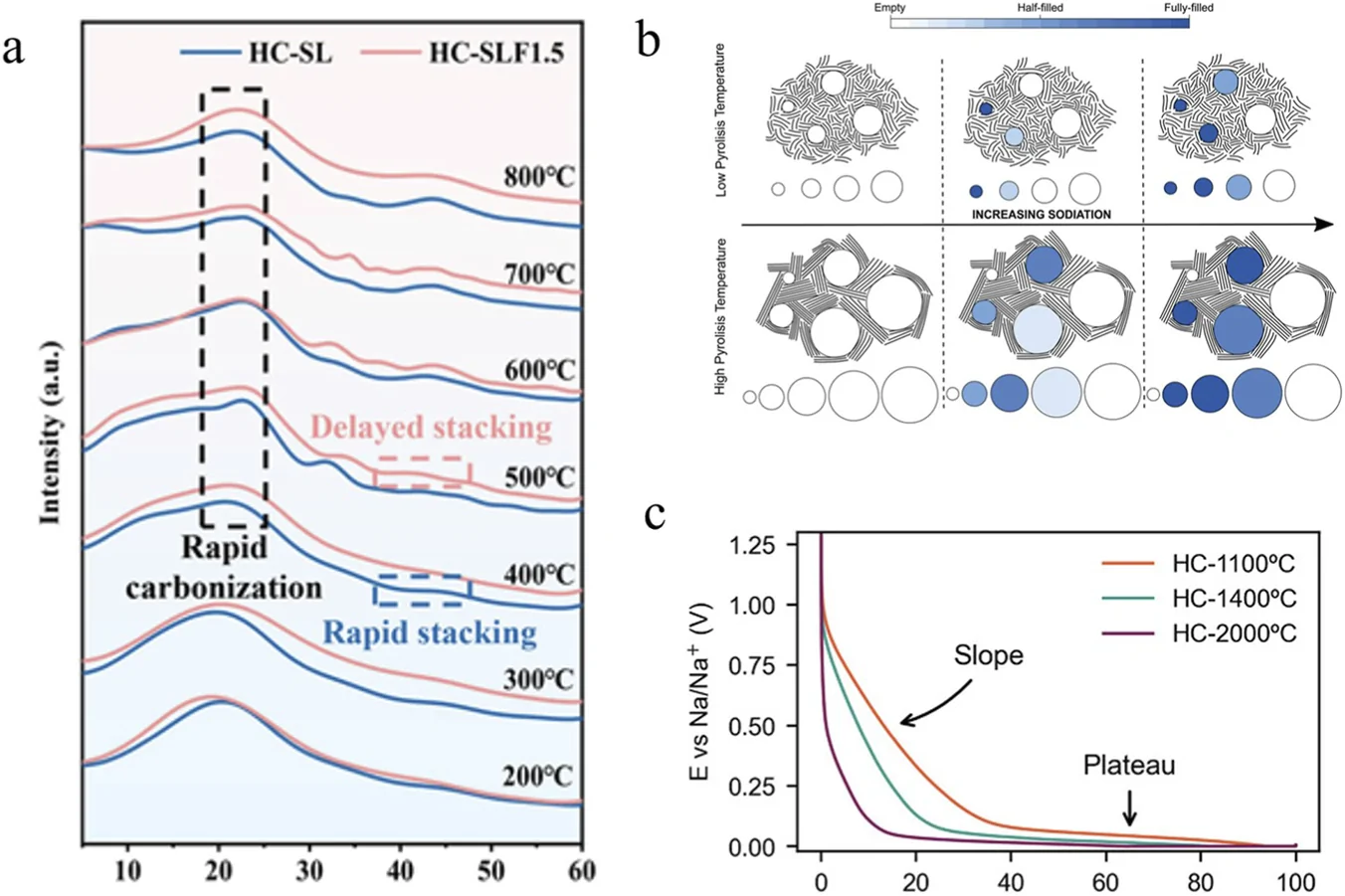
**(a)** XRD patterns at different carbonization temperatures. **(b)** Schematic representation of the microstructure and the storage of sodium ions in closed pores for HCs synthesized at different pyrolysis temperatures. **(c)** Normalized capacity-potential curves of the different HC structures (Iglesias et al., 2023).

#### 2.3.2 Precursor selection

The intrinsic molecular properties of precursors—crosslinking density, oxygen content, and aromaticity—define the boundary conditions for carbonization, governing carbon skeleton rearrangement pathways and graphitization resistance during thermal treatment. Three primary precursor categories follow distinct design rationales. Biomass precursors tune porosity via the divergent pyrolysis behaviors of lignocellulosic components, where the cellulose-to-lignin ratio acts as a molecular-level lever to balance open and closed porosity and tailor sodium storage performance. As a highly controllable synthetic system, phenolic resin achieves elevated closed-pore fraction and initial Coulombic efficiency through pre-oxidation-enhanced crosslinking. Pitch precursors, despite outstanding cost advantages, require oxygen crosslinking pretreatment to suppress their inherent graphitization tendency and induce closed-pore nucleation. The detailed mechanistic insights, regulatory approaches, and performance optimization of these precursor systems will be systematically elaborated in Section 3.

#### 2.3.3 Heteroatom doping

Heteroatom doping constitutes atomic-scale chemical modification of the carbon skeleton, introducing local electronic perturbations and geometric distortions that simultaneously influence interlayer spacing, Na^+^ adsorption energetics, defect density, and electronic conductivity. The effect of each dopant element is mechanistically distinct.

Nitrogen doping, particularly in the form of pyrrolic-N and pyridinic-N configurations at carbon-edge sites, introduces electron-rich Lewis basic centers that enhance Na^+^ adsorption via Faradaic pseudocapacitance, contributing additional sloping-region capacity above 0.1 V, while quaternary-N substitution within the graphene plane improves bulk electronic conductivity (Yang Y. et al., 2025). Sulfur doping, owing to sulfur’s larger covalent radius (105 p.m. vs. 77 p.m. for carbon), expands the interlayer spacing by 0.01–0.03 nm and simultaneously forms C–S–C covalent bridges between adjacent carbon layers that resist graphitization-induced layer collapse during high-temperature carbonization (Liu et al., 2025). Earlier, Li and others (Li et al., 2017) improved sodium storage performance by doping three atoms (Figure 5). The interplay between intrinsic carbon defects and extrinsic dopant sites has recently been shown to be critical: N/S co-doped hard carbons exhibit a nonlinear synergistic enhancement in total capacity that exceeds the sum of single-dopant contributions, because S-expanded interlayers lower the Na^+^ diffusion barrier while adjacent N sites enhance the local electron density of the carbon layer, strengthening electrostatic binding of Na^+^—the spatial proximity of the two dopant types enables a single carbon-layer region to benefit simultaneously from reduced diffusion resistance and increased sodium affinity (Zhao et al., 2026). Zhang is a relatively new method, which uses covalent organic framework (COF) as precursor to obtain porous carbon by high temperature carbonization, and uses nitrogen (N) and sulfur (S) co-doping to expand the carbon layer spacing and introduce defect active sites (Zhang et al., 2022). Phosphorus doping provides a complementary lever: the P–C bond, with phosphorus’s still larger covalent radius, both expands the carbon interlayer and, through P–O–C bridge formation, promotes closed-pore nucleation during carbonization by retarding carbon-layer stacking (Chen et al., 2025). Boron doping, in contrast, introduces electron-deficient sites that modulate the curvature of carbon layers during thermal treatment, guiding the formation of twisted pseudo-graphitic microcrystallites that provide short-range Na^+^ transport channels favorable for high-rate performance (Zhang et al., 2025b). Silicon doping has recently emerged as another effective strategy to expand interlayer spacing while simultaneously reducing pseudo-graphitic crystallite size, synergistically lowering the Na^+^ solid-state diffusion barrier (Lin Y. et al., 2026). Oxygen functional groups, particularly C=O carbonyls, provide additional sloping-region Na^+^ adsorption sites but also contribute to irreversible electrolyte decomposition at low potentials, creating a fundamental trade-off between sloping capacity enhancement and initial Coulombic efficiency (Li X. et al., 2025). The emerging consensus is that the net electrochemical outcome of heteroatom doping depends not only on the dopant element type and content but critically on the structural context of the carbon matrix—the same dopant species may function as a beneficial active site in a highly defective low-temperature carbon yet become a detrimental defect trap in a highly ordered high-temperature carbon—and that rationally designed co-doping, in which two or more dopants with complementary functions (e.g., S for interlayer expansion, N for electronic activation, P for closed-pore promotion) are introduced simultaneously, offers the most promising route to synergistic capacity enhancement.

**FIGURE 5:**
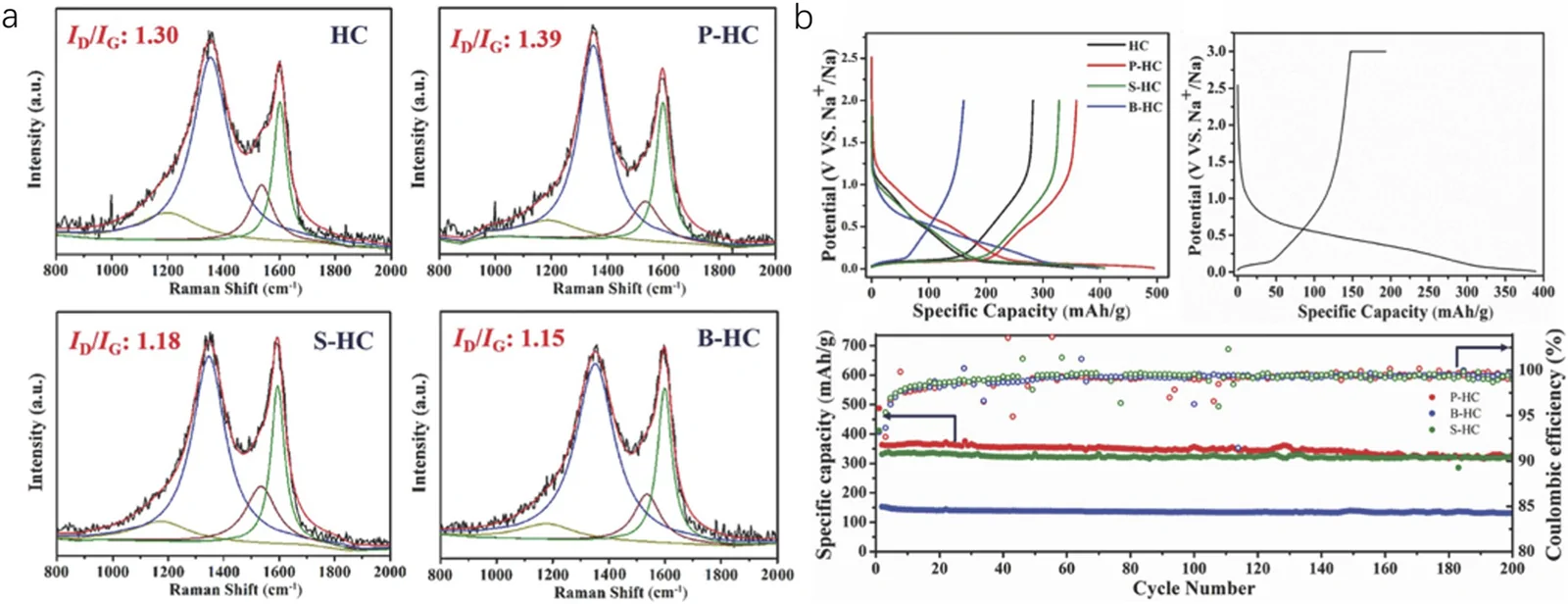
Effect of heteroatom doping: **(a)** Raman spectra of different carbon samples, which are deconvoluted into four Lorentzian peaks: TPA (dark yellow), D (blue), A (wine), and G (green). HC, P-HC, S-HC, and B-HC; **(b)** Electrochemical performance of hard carbons in C/Na half-cells (Li et al., 2017).

#### 2.3.4 Pore structure and specific surface area

Pore structure represents the terminal product of all precursor selection, carbonization, and doping parameters, and constitutes the structural interface that directly communicates with electrochemical performance—most critically, initial Coulombic efficiency (ICE) and plateau capacity. The fundamental design challenge lies in the open-pore/closed-pore trade-off (Figure 6): open micropores with high specific surface area provide abundant Na^+^ adsorption sites that contribute sloping-region capacity, but their extensive electrode–electrolyte interface drives irreversible solid-electrolyte interphase (SEI) formation that consumes sodium inventory and depresses ICE below 60% (Ren et al., 2026); closed nanopores, by contrast, are inaccessible to electrolyte and instead host quasi-metallic sodium cluster filling at low potentials, contributing plateau capacity below 0.1 V without the irreversible surface-reaction penalty (Gan et al., 2025; Zhang et al., 2025c). The formation of closed pores is intimately tied to carbon-layer curvature: during carbonization, the release of volatile species and the thermally activated rearrangement of carbon layers induces localized bending and folding that seals open pore apertures (Wu et al., 2026), converting interconnected micropore networks into discrete closed nanopores. The population and size distribution of closed pores can be tuned by controlling the rate and temperature window of this curvature-driven closure process—for instance, step-temperature strategies that decouple volatile release from carbon-layer rearrangement have been shown to increase closed-pore volume while suppressing excessive open porosity (Li H. et al., 2025). The ideal pore architecture for hard carbon anodes must simultaneously satisfy four constraints: a low open-pore fraction (ensuring ICE above 85%), a high closed-pore volume (providing plateau capacity above 200 mAh g^-1^), a pore size distribution centered at 1–2 nm (accommodating 2–3 layers of quasi-metallic sodium clusters), and a moderate defect density in the pore-wall carbon layers (maintaining Na^+^ transport channels between intercalation sites and closed pores) (Yang et al., 2026). Post-synthesis pore-closure strategies, such as chemical vapor deposition (CVD) of a thin carbon overlayer that selectively blocks open-pore apertures without affecting internal closed-pore volume, have demonstrated the ability to raise ICE from below 60% to above 85% while largely preserving plateau capacity (Zhang et al., 2026a; Zhou Q. et al., 2026). Soft-carbon coating represents another effective co-regulation strategy, simultaneously modifying the surface chemistry and sealing surface-connected pores of hard carbon, which markedly improves high-rate sodium storage (Zhang et al., 2026b). Despite this efficacy, CVD-based pore sealing remains difficult to translate to industrial scale: vapor-phase deposition carries a high processing cost, and achieving uniform coating across large-scale powder batches is a persistent challenge. These practical limitations—further elaborated in the industrialization discussion of Section 4—currently confine such post-treatments mainly to laboratory-scale demonstration. More recently, sequential pore-creation-and-sealing approaches have been developed that first generate a well-defined ultramicroporous template through controlled activation and then seal the pore apertures through a secondary carbonization treatment, decoupling pore formation from pore closure and enabling independent optimization of closed-pore volume and surface area. Chemical conversion routes offer a complementary pore-closure path: tuning porous carbon into hard carbon through an substitution reaction has been shown to reorganize the carbon framework into a closed-pore-rich architecture, enhancing plateau-region sodium storage (Zhou W. et al., 2026). Achieving this multi-constraint pore-architecture equilibrium requires synergistic optimization of precursor chemistry, carbonization temperature profile, and post-treatment strategy.

**FIGURE 6:**
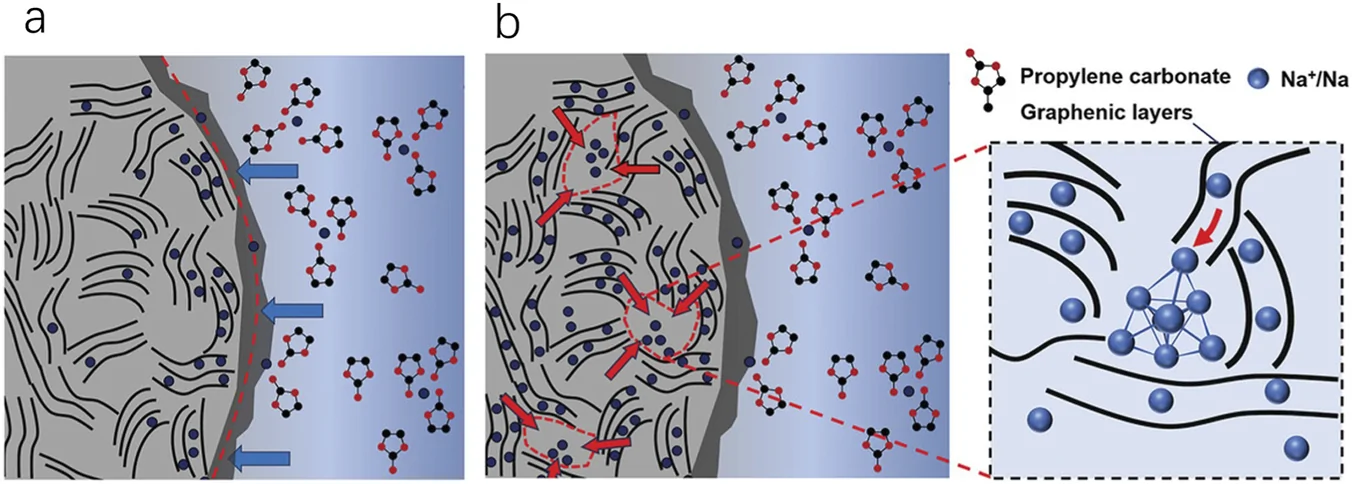
**(a)** Schema on diffusion in hard carbon particle for insertion into pseudo-graphitic domains and **(b)** inside the carbon into the pores. Dashed red lines illustrate the diffusion front for intercalation and pore filling (Aniskevich et al., 2024).

## 3 Classification of precursors for hard carbon

### 3.1 Biomass precursors

Biomass is the preferred feedstock for hard carbon anodes because it is cheap, abundant, and naturally rich in heteroatoms and hierarchical pores. Recent work has largely converged on three classes of biomass—agricultural waste, plant natural polymers, and lignocellulose—each of which raises different questions and calls for different strategies (Zhang et al., 2025d).

Agricultural waste is attractive mainly for its price and availability, but it is also a variable, poorly defined feedstock, so the recurring question is how to turn such raw material into carbon with a controlled microstructure. One answer is to regulate the carbonization step itself. Bagasse pyrolyzed along a temperature gradient (Figure 7) passes through successive storage regimes—adsorption, intercalation, and pore filling—that reinforce one another rather than compete, and this is the basis of the carbon reported by Kuang et al. (2026), which combines high reversible capacity with stable cycling. A second line of thinking places the pores, not the heating profile, at the center. In corn-cob-derived carbon, fast-charging performance is governed mainly by the size and wall thickness of the closed pores, and by tuning these two parameters directly, Zhang et al. (2026c) worked out the quantitative relationship between closed-pore geometry and rate capability. Pore design alone, however, does not solve the heterogeneity problem, since no two batches of agricultural residue are the same. Cui J. et al. (2026) took a data-driven route instead, building a machine-learning-guided carbonization framework in which Joule heating supplies a programmable thermal profile, so that crop straw, cotton stalks, and other residues are all converted into high-performance hard carbon through a single process. Capacity can also be pushed beyond what the carbon skeleton itself can store: in a hard carbon host derived from the invasive plant Canadian goldenrod, embedded bismuth nanoparticles contribute their alloying reaction alongside the carbon’s sodium storage, adding capacity on top of the matrix (Shen et al., 2026a).

**FIGURE 7:**
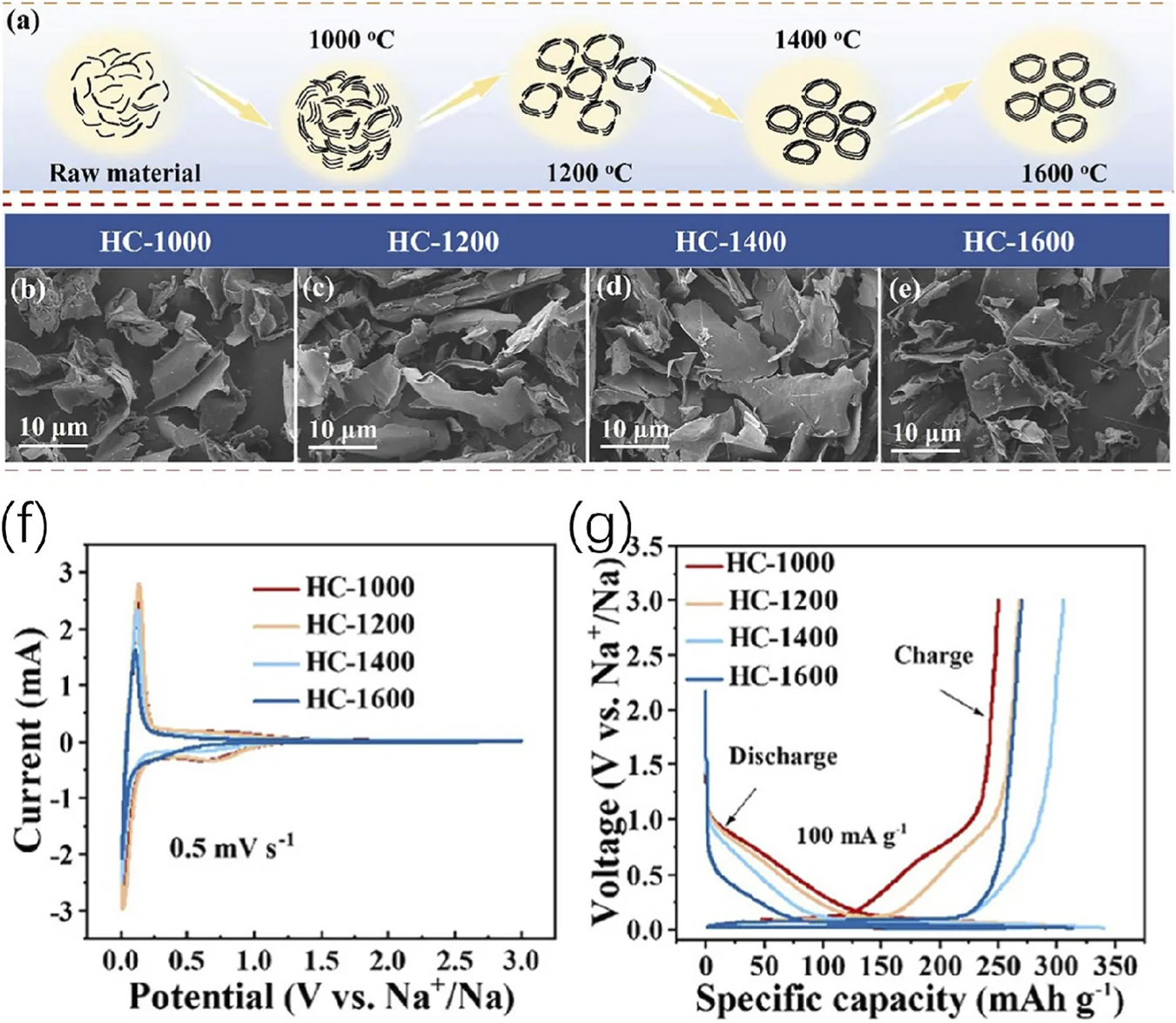
**(a)** Schematic illustration of structural evolution of bagasse-derived hard carbons with rising carbonization temperature **(b–e)** SEM images of the as-synthesized samples. **(f)** Initial CV curves and **(g)** initial GCD curves of the resultant electrodes (Kuang et al., 2026).

Plant natural polymers present the opposite situation (Xu et al., 2018). Molecules such as starch and cellulose have well-defined, repeatable structures, so the design freedom moves upstream to the molecule itself, and the goal becomes programming the pore structure before carbonization rather than coaxing it out of an ill-defined feedstock. Wen Z. et al. (2025) proposed a molecular suturing strategy, crosslinking polysaccharide chains into a network before pyrolysis so that the network, not chance, dictates where uniform closed pores form; the carbon thereby attains an unusually high low-voltage plateau capacity. The same end can be approached from the composition side. Removing oxygen—and with it the volatiles that escape and disrupt the skeleton during heating—keeps the structure intact while raising carbon yield, the principle behind a dehydration-assisted synthesis route (Xu et al., 2026), and a related strategy that uses cellulose and eliminates volatile components at the source suppresses the disordered pores that would otherwise form, giving a markedly higher first-cycle Coulombic efficiency (Huang Y. et al., 2025). These studies together point to a simple rule: the crosslinking density and the placement of functional groups in the precursor molecule ultimately determine the orientation of the pseudographitic domains and the connectivity of the closed pores, so tuning them at the molecular level offers a direct handle on the final carbon (Xu H. et al., 2025).

Lignocellulose is not a single material but a composite of cellulose, hemicellulose, and lignin, and the three components respond very differently to heat, a difference that underlies both the difficulty and the opportunity of this class. Regarding how the initial morphology (morphology) of the cellulose precursor affects the microstructure and sodium storage performance of the final hard carbon anode Sun et al. Sarma et al. (2024). Selected three precursors with different morphologies and carbonized under the same conditions. The relationship between the precursor morphology and the final structure of hard carbon was revealed by means of Raman spectroscopy and XRD. Since it is lignin, with its crosslinked aromatic structure, that mainly evolves into closed pores during carbonization, an obvious move is to protect it: pre-oxidizing wood to selectively stabilize the lignin component keeps the closed pores from collapsing as the carbon develops (Dai et al., 2026). Another approach is to link the three components together so they evolve in a coordinated fashion, and Wu F. et al. (2025) showed that chemical bridges built among cellulose, hemicellulose, and lignin can steer the orientation and size of the pseudographitic domains. The underlying mechanism has since been spelled out in more detail, with Zhang et al. (2025e) tracing how the differential pyrolysis of the three components jointly governs closed-pore formation and pseudographite-domain evolution. Once the component-level behavior is understood, it can be put to use directly: Tan et al. (Ren et al., 2025) found that ultramicropore defects, rather than being an unwanted byproduct, can be converted *in situ* into closed pores when elemental regulation is combined with defect engineering, raising the plateau capacity in a controlled way.

### 3.2 Sugar precursors

Sugar precursors such as starch and glucose feature well-defined molecular structures, high carbon yields, and high amenability to chemical modification, making them an ideal model system for the rational microstructure design of hard carbon materials. Substantial progress has been achieved in this research field in recent years, particularly in the domains of crosslinking chemistry and pore structure regulation.

In chemical crosslinking regulation, Zhang et al. (2026d) used glucose as the raw material and constructed highly crosslinked precursors via intermolecular hyper-crosslinking with resorcinol. After carbonization, abundant uniform closed pores are generated *in situ* within the carbon matrix, delivering high plateau capacity. The enzyme-mediated crosslinking strategy (Zhang et al., 2026e) adopts a distinct approach: it replaces traditional chemical crosslinkers with bioenzyme catalysis to achieve precise crosslinking of starch molecular chains under mild conditions, avoiding the adverse effects of excessive crosslinking on the carbon skeleton microstructure. Oxygenation crosslinking (Wang et al., 2025a) further couples oxidation treatment with crosslinking, and simultaneously constructs carbonyl interfaces and closed-pore structures in starch-derived carbons. Carbonyl groups provide sodium adsorption sites for the sloping capacity region, while closed pores contribute to the low-voltage plateau capacity, realizing coordinated capacity enhancement in both the sloping and plateau regions. Crosslinking degree regulation strategies (Yu et al., 2026) take crosslinking density as the core parameter, and systematically modulate the crosslinking level of starch to optimize closed-pore abundance and size distribution, achieving an effective balance between plateau capacity improvement and rate capability maintenance.

In microstructure engineering, Shen et al. (2026b) proposed an air stabilization strategy (Figure 8), which stabilizes the spherical morphology of starch via simple hot-air oxidative crosslinking without additional reagents. After carbonization, regular spherical closed pores are formed, enabling a balance between high capacity and high initial Coulombic efficiency. The phosphorus doping synergistic strategy (Shen et al., 2026c) incorporates phosphorus atoms into the starch framework. By virtue of the large covalent radius of phosphorus atoms, this strategy expands the carbon interlayer spacing and promotes closed-pore nucleation, realizing dual regulation of interlayer spacing and closed-pore formation. Oxygenation-driven regulation strategies (Zhang et al., 2025f) precisely control the oxidation degree of starch to tailor the growth orientation of pseudographite domains and the formation rate of closed pores during carbonization, opening up new avenues for microstructure regulation. Xie et al. (2026) systematically revealed that ultramicropores in starch-derived hard carbon undergo *in situ* fusion and transformation during the heating process, elucidating the structural evolution mechanism and physical origin of closed-pore formation. This finding provides critical insights for the rational design of closed-pore evolution pathways based on ultramicropore transformation.

**FIGURE 8:**
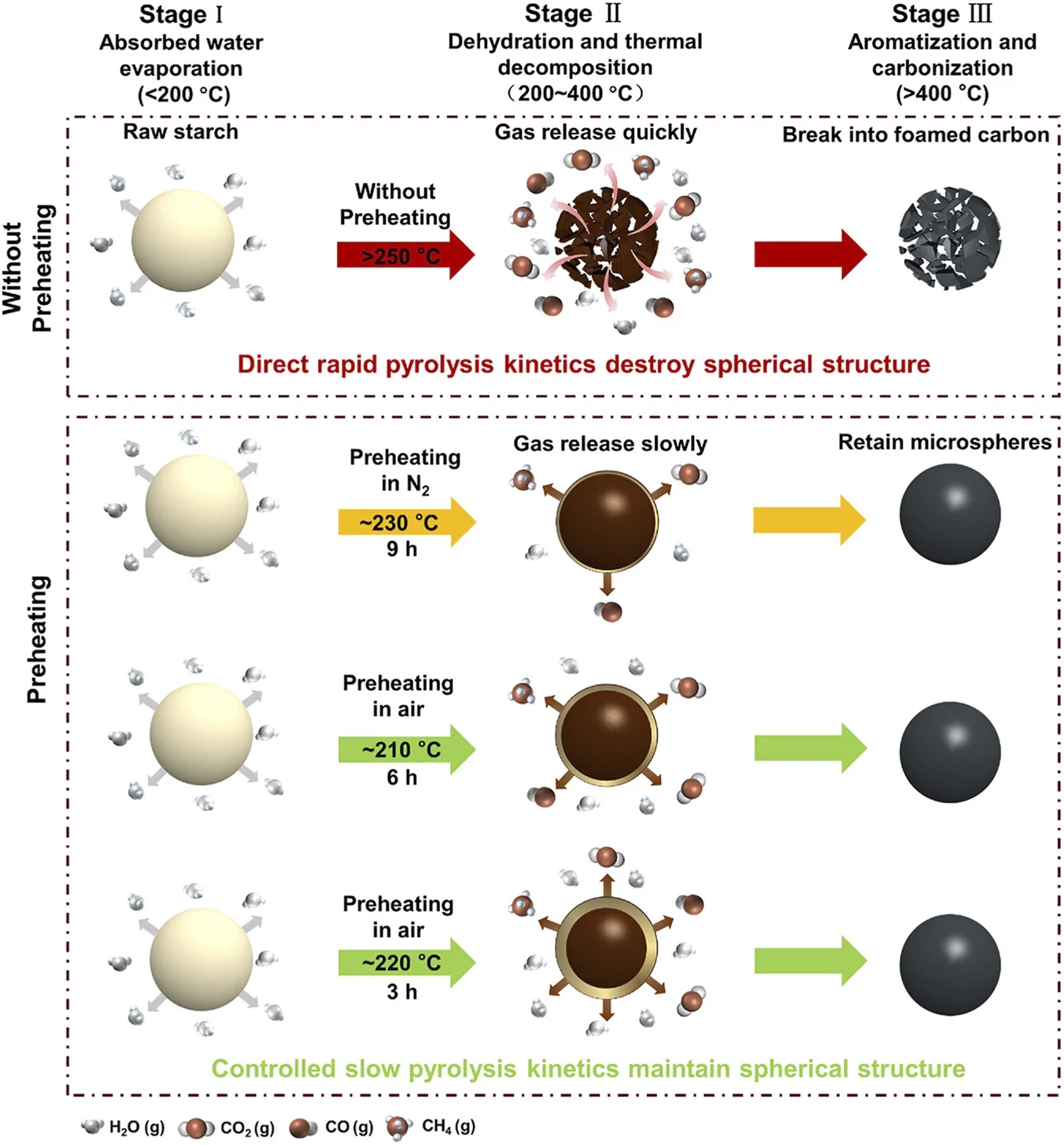
The proposed reaction mechanism for the conversion of starch to hard carbon. Starch without preheating undergoes rapid pyrolysis when the temperature exceeds 250 °C, releasing a large amount of gas, which leads to particle pulverization. However, preheating (such as 230 °C for 9 h in N_2_, 210 °C for 6 h in air, and 220 °C for 3 h in air) enables slow release of small-molecule gases, which helps retain the microsphere morphology (Shen et al., 2026b).

### 3.3 Resin-based precursors

Resin-based precursors have strong molecular structure design flexibility, high carbon yield, and controllable purity, making them hard to resist. It has unique advantages in the rational structural regulation of carbon. In recent years, research has focused on phenolic resin as the main component, expanding to blend systems such as urea-formaldehyde resin, focusing on crosslinking chemistry and interstructure and pyrolysis pathways, achieving fine regulation of carbon microstructure (Zhou et al., 2025).

Phenolic resin is a different kind of precursor from biomass: its structure can be designed before carbonization, so the question is less about taming a variable feedstock than about choosing which design lever to pull. One lever is geometric. In phenolic-derived carbon, closed pores do not appear at random—they inherit their positions from the three-dimensional packing of the prepolymer, and Guo et al. (2025) showed that directing this packing controls both where the pores form and how large they grow, strengthening the low-potential plateau capacity. A second, chemical lever is crosslink density. Curing the resin to a controlled degree of crosslinking lowers the defect density of the carbon skeleton while building a three-dimensional conductive network; Xiong et al. (2026) used this to improve cycling stability without losing rate capability. Lin J. et al. (2026) pulled a third lever, the aggregation state of the chains, reasoning that strong aggregation in the precursor locks in an unfavorable carbon-layer arrangement—loosening it produced interconnected closed pores that serve as both sodium storage sites and ion-migration channels. Co-doping is a fourth lever, acting on geometry and electronics at once: two heteroatoms of different atomic size widen the interlayer spacing and induce closed-pore nucleation, giving the large capacity gain reported by Chen et al. (2025). These levers are not confined to phenolic chemistry—modifying urea-formaldehyde resin with phenol preserves the structural integrity of the carbon skeleton and offers a practical route to low-cost resin systems beyond phenolic (Wang et al., 2026).

The design of general-purpose resin precursors has progressed along a clear logical trajectory: from mechanistic understanding of pyrolysis to predictive structure–property correlation, and from there to *in situ* intervention during synthesis. Early studies traced the entire pyrolysis pathway, following resin precursor molecules through their intermediate species to the terminal carbon, and thereby established the intrinsic relationship between precursor molecular structure and final microstructure—a relationship that provides the theoretical foundation for forward precursor design (Wen et al., 2026). Building on this mechanistic understanding, Zhang et al. (2026f) translated it into a quantitative structure–property correlation, demonstrating that a single molecular parameter, namely, the conformation of the precursor chains, simultaneously determines the orientation of the pseudo-graphitic domains and the aspect ratio of the closed pores in the resulting carbon. Once such a correlation became available, the control point naturally shifted from post-carbonization adjustment to the polymerization stage, where molecular-level polymerization and crosslinking are coupled so that a preset crosslinked network is constructed *in situ* during resin synthesis, allowing an optimized microstructure to develop directly upon carbonization without any post-treatment (Li S. et al., 2025). This logic has been extended one step further: Yuan et al. (Li L. et al., 2026) eliminated exogenous crosslinkers altogether, exploiting the intrinsic reactive functional groups of the resin for both intramolecular and intermolecular crosslinking, thereby avoiding impurity introduction while preserving purity and structural controllability.

### 3.4 Asphalt-based precursors

Pitch, a by-product of coal chemical processing and petroleum refining, combines high carbon content, tunable aromaticity, and low cost—attributes that make it the most economically viable precursor for the large-scale production of hard carbon anodes. These same attributes, however, impose an intrinsic constraint: high aromaticity together with low oxygen content predisposes pitch toward graphitization, so that conventional carbonization naturally yields soft carbon rather than hard carbon. The central challenge in pitch-based systems is therefore to override this tendency and redirect carbonization toward a disordered, closed-pore-rich microstructure, a goal that recent research on coal-tar pitch has pursued along three dimensions—pre-oxidation, crosslinking chemistry, and molecular reconstruction.

Intervention along the first dimension can occur at different levels of the transformation process. At the molecular level, the aromatic lamellae themselves constitute the controlling unit: their size distribution and spatial arrangement jointly govern the balance between the thermodynamic stability of the carbon framework and the kinetics of sodium storage, and Li L. et al. (2026) showed that regulating these parameters—the essence of molecular reconstruction—allows the two to be finely balanced, yielding simultaneous optimization of high capacity and fast charge–discharge. At the process level, the carbonization regime provides an independent lever: precise control of low-temperature carbonization restricts domain growth, constructing short-range ordered, few-layer pseudo-graphitic domains that shorten the solid-state diffusion path of sodium ions and supply abundant short-range transport channels for ultrafast charge–discharge (Wang et al., 2025b). At the chemical level, pretreatment and elemental doping can be coupled to act jointly on the interlayer region: during pre-oxidation, iodine molecules intercalate between the pitch layers and form charge-transfer complexes that enhance the degree of oxidative crosslinking while promoting closed-pore nucleation in the subsequent carbonization, producing an outstanding contribution to the low-potential plateau capacity (Wei et al., 2025). Oxidation-induced molecular structural transformation has further been shown to depend sensitively on the softening point of the pitch feedstock, providing a tunable handle for controlling the crosslinking degree and, in turn, the sodium-storage-active microstructure (Yang W. et al., 2025).

In the chemical dimension, the strategies developed for pitch-derived carbon all converge on a single objective: to suppress graphitization while simultaneously constructing sodium-storage-active structure. The most direct route operates on the aromatic layer itself, coupling two complementary elements—sulfur, whose intermolecular crosslinking forms C–S–C bridge bonds that restrain aromatic-ring rearrangement during carbonization, and phosphorus, whose larger atomic radius expands the carbon layers and introduces additional sodium storage sites; acting together, they markedly enhance overall capacity (Han et al., 2026). A more delicate approach concerns the doping stoichiometry: the N/P ratio must be tuned so that the gain in active sites is not offset by defect traps, raising sloping-region capacity while avoiding irreversible capacity loss (Kang et al., 2026). Beyond the carbon layers, rigid bridges can be introduced between aromatic molecules through co-crosslinking of urea-formaldehyde resin with coal-tar pitch, a strategy that effectively suppresses the graphitization tendency and converts low-cost coal pitch into high-performance hard carbon (Zhang et al., 2026g). Pore architecture offers yet another control route: *in situ* melt activation by alkali-metal salts during carbonization constructs a loose closed-pore framework within the pitch-derived carbon, circumventing the excessive open porosity and low yield associated with conventional KOH activation and thereby balancing high plateau capacity against carbon yield (Wen C. et al., 2025). Extending from coal-tar to petroleum pitch, attention shifts from *post hoc* engineering to *a priori* prediction: molecular descriptors—aromaticity, alkyl chain length, and heteroatom content—derived from the feedstock’s composition (Figure 9) can be quantitatively correlated with the closed-pore parameters after carbonization, enabling carbon-microstructure prediction and forward design directly from raw-material characteristics (Cui Y. et al., 2026). Ultimately, these bulk-phase strategies must be reconciled with the electrode–electrolyte interface: a dual structure–interface regulation strategy optimizes both the ordered closed-pore structure in the bulk phase—to raise plateau capacity—and the surface functional groups at the interface—to induce uniform SEI formation—achieving a synergistic improvement in sodium storage capacity and long-cycle stability (Xu X. et al., 2025). A comparative classification and performance summary of each precursor class is presented in Table 2.

**FIGURE 9:**
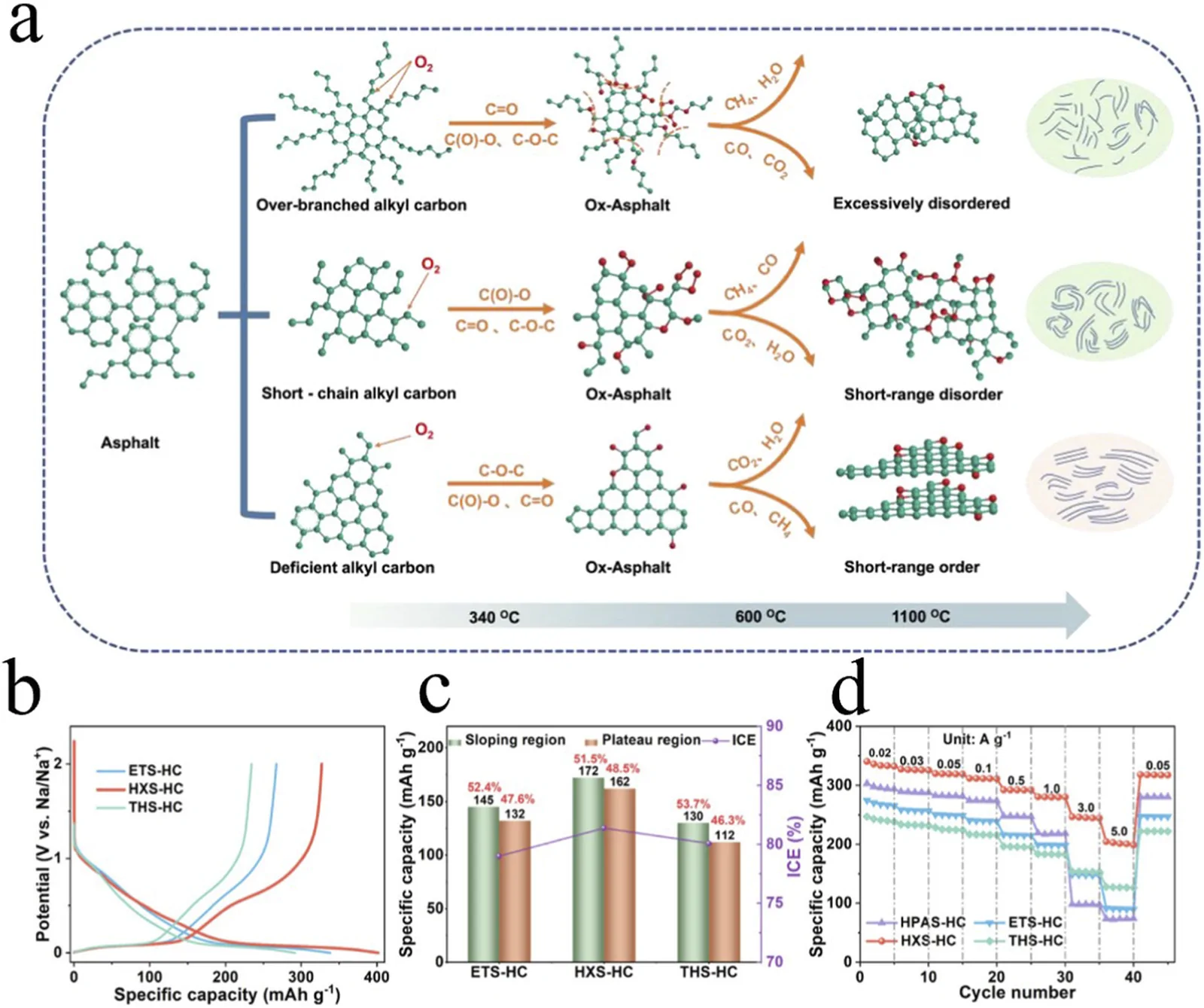
**(a)** Proposed oxidation mechanism and structural evolution pathway, linking aliphatic side-chain reactivity to crosslinking, defect generation, and stacking regulation. **(b)** First cycle charge/discharge curve at a current density of 50 mA g^−1^. **(c)** The ICE, slope and plateau region contribution. **(d)** The rate performance of the HPAS-HC, ETS-HC, HXS-HC, and THS-HC at different currents from 20 mA g^−1^ to 5 A g^−1^ (Cui Y. et al., 2026).

**TABLE 2:** Performance table for each precursor classification.

Precursor	Carbon yield (wt%)	Crosslinking density	Specific capacity (mAh g^−1^)	Remarks
Phenolic resin (resole)	50–60	High (methylene + ether-bond crosslinked network)	280–350	Most classical precursor; thermosetting; high carbon yield; high plateau capacity fraction (Li J.-H. et al., 2026)
Epoxy phenol novolac resin	45–55	High (epoxy ring-opening crosslinking)	300–360	Multiple epoxy groups provide high crosslink-point density; tunable interlayer spacing (Fan et al., 2023)
Epoxy resin (general)	40–50	Medium-high (amine/anhydride curing)	250–320	Synergistic enhancement when composited with anthracite (Wang Q. et al., 2024)
Polyacrylonitrile resin (PAN)	38–48	Medium (pre-oxidative cyclization/dehydrogenation)	250–320	Requires pre-oxidation stabilization; electrospinning for nanofiber morphology (Wang et al., 2025c)
Furfural/Furfuryl Alcohol resin	45–55	Medium-high (acid-catalyzed polycondensation)	260–330	Biomass-derived, renewable; can produce spherical carbon (Zhou et al., 2016)
Sodium lignosulfonate-formaldehyde resin	38–48	Medium (sulfonate + methylol crosslinking)	270–320	Industrial byproduct utilization, low cost; S-containing groups introduce defects (Moctar et al., 2025)
COPNA resin	50–65	High (3D aromatic ring crosslinked network)	300–380	3D-structured precursor; superior low-voltage plateau behavior (Pu et al., 2026)
Polyaniline (PANI)	30–42	Low-medium (chain rigidity + hydrogen bonding)	250–340	N self-doping provides pseudocapacitive contribution and extra active sites (Huo et al., 2025)
Ion-exchange resin (polystyrene sulfonate type)	25–38	Medium (divinylbenzene crosslinking)	220–300	Pre-load metal ions (K^+^/Na^+^) as pore-forming agents; tunable pore structure (Hou et al., 2025)

### 3.5 Comparative assessment of precursor classes

Taken together, the four precursor classes occupy distinct positions in the cost–controllability–performance landscape. Biomass precursors are the most economical and sustainable, but their heterogeneous and variable composition limits batch-to-batch consistency; sugar-derived carbons offer the most uniform and tunable microstructure with high initial Coulombic efficiency, at the cost of a more expensive feedstock; resin precursors provide the greatest structural controllability through molecular design and crosslinking, yet remain cost-constrained for large-volume applications; and pitch precursors deliver the lowest cost with the highest carbon yield, but require deliberate pre-oxidation, crosslinking, or molecular reconstruction to overcome their intrinsic graphitization tendency. In practice, the choice of precursor is therefore scenario-dependent: cost-sensitive grid-storage applications favor biomass and pitch routes, whereas power- and high-end applications—where rate capability and consistency dominate—tend toward sugar- and resin-derived carbons. This comparative perspective is synthesized in Table 1 and further elaborated in the industrialization analysis of Section 4 (Yang W. et al., 2025; Yuan et al., 2025).

## 4 Progress in hard carbon commercialization

### 4.1 Market trends for hard carbon used in sodium-ion batteries over the past decade

As early as 2000, Stevens and Dahn reported the reversible sodium storage behavior of glucose pyrolysis hard carbon in sodium-ion batteries, with a reversible capacity close to 300 mAh g^-1^, laying the academic foundation for hard carbon as a sodium anode (Ste et al., 2000). However, over the following decade, overall research enthusiasm for sodium-ion batteries was limited, hard carbon materials remained at the laboratory level for a long time, with only Japan’s Kuraray supplying small batches of plant-based hard carbon under the “Kuranode” brand to global research institutions, with per-ton prices consistently above 300,000 RMB, far from meeting the economic premise for industrialization.

The real turning point came in 2021. In July of that year, CATL officially launched its first-generation sodium-ion battery, adopting a layered oxide cathode and hard carbon anode technology route, with a cell energy density of 160 Wh kg^-1^. This incident has pushed hard carbon from a laboratory consumable to the core focus of the industry. Since then, the commercialization process of hard carbon anode materials in China has accelerated rapidly. In 2022, Shanshan Co., Ltd. took the lead in building China’s first thousand-ton hard carbon production line and achieving mass supply, followed closely by companies like Baisige and Shengquan Group. By the end of 2023, domestic hard carbon shipments had surpassed the 1,000-ton level, and Kuraray’s market share in China quickly dropped from over 90% of the nearly monopoly to less than 40%.

In terms of precursor technology routes, domestic companies have taken a path completely different from Japan. Kuraray has long relied on carefully selected coconut shell raw materials, exchanging extremely high craftsmanship precision for product consistency, but the costs are high and capacity expansion is slow. Bestger adopts a four-route parallel strategy: biomass-based (starch/coconut shell), coal-based, resin-based, and asphalt-based. Its high-end products are expected to exceed 400 mAh g^-1^ in reusable capacity by 2025, with Coulombic efficiency stabilizing above 90% for the first time, and performance indicators already on par with international benchmarks. Shengquan Group, relying on agricultural waste such as straw as a precursor (Figure 10), built China’s first 10,000-ton biomass hard carbon mass production line in 2024, reducing the cost per ton to around 40,000 yuan—only a quarter of Kuraray’s price during the same period. Huang et al. pointed out that the natural porous structure of biomass precursors closely matches the “adsorption-embedding-pore filling” mechanism of hard carbon sodium storage, making it an ideal path to achieve low-cost, high-capacity hard carbon (Hao et al., 2026).

**FIGURE 10:**
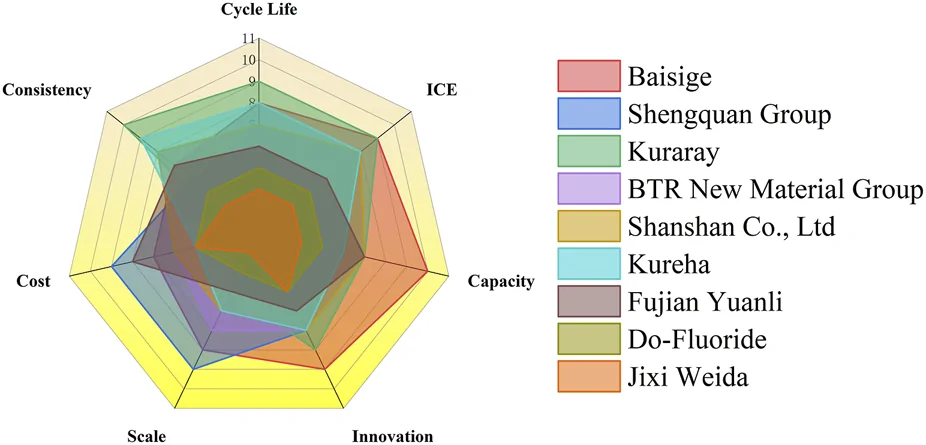
Performance radar chart of major hard carbon companies.

From a price perspective, the unit price of hard carbon fell from 300,000350,000 yuan ton^−1^ (Kuraray lab-grade) in 2015, then accelerated after domestic substitution began in 2022. The industry average price dropped to about 50,000 yuan ton^−1^ in 2025, and further approached 40,000 yuan ton^−1^ in 2026. Coal-based and straw-based routes are expected to reach 30,000 yuan ton^−1^ around 2027–2028—the threshold for parity with lithium battery graphite anodes.

Compared to the flourishing domestic market, the commercialization pace of hard carbon overseas is clearly lagging behind. Although Japan’s Kureha and China’s BTR have established a joint venture factory in Ningbo, their production capacity is less than 10,000 tons. India’s Epsilon Advanced Materials and Canada’s E-Power Resources will only first issue or obtain hard carbon-related patents in 2026, still far from large-scale production. Overall, China has achieved comprehensive leadership in the industrialization scale, cost control, and technological integrity of hard carbon anode materials, and the global focus of the hard carbon supply chain is irreversibly shifting toward China.

### 4.2 Predictions and projections in hard carbon commercialization

In terms of capacity, 400 mAh g^-1^ is approaching the theoretical upper limit of hard carbon (about 420–450 mAh g^-1^), further driving the diminishing marginal gains of higher capacity. The industry will split into two product lines: energy storage scenarios anchoring 330–350 mAh g^-1^ balancing cost and cycle life, while power and high-end scenarios maintain 380–400 mAh g^-1^ focusing on efficiency and rate utilization.

On the cost side (Figure 11; Table 3), raw material costs for coal-based and straw routes have approached zero, and the focus of cost reduction is shifting to manufacturing—continuous kilns are replacing batch equipment, and single-line capacity is rising to 30,000–50,000 tons. Leading companies’ total cost per ton is expected to reach 25,000–30,000 yuan around 2028, at which point hard carbon and lithium battery graphite anodes will reach parity. However, the scale effect barrier at the 10,000-ton level is steep, and second-tier companies that fail to secure long-term contracts before 2027 will be cleared out more rapidly.

**FIGURE 11:**
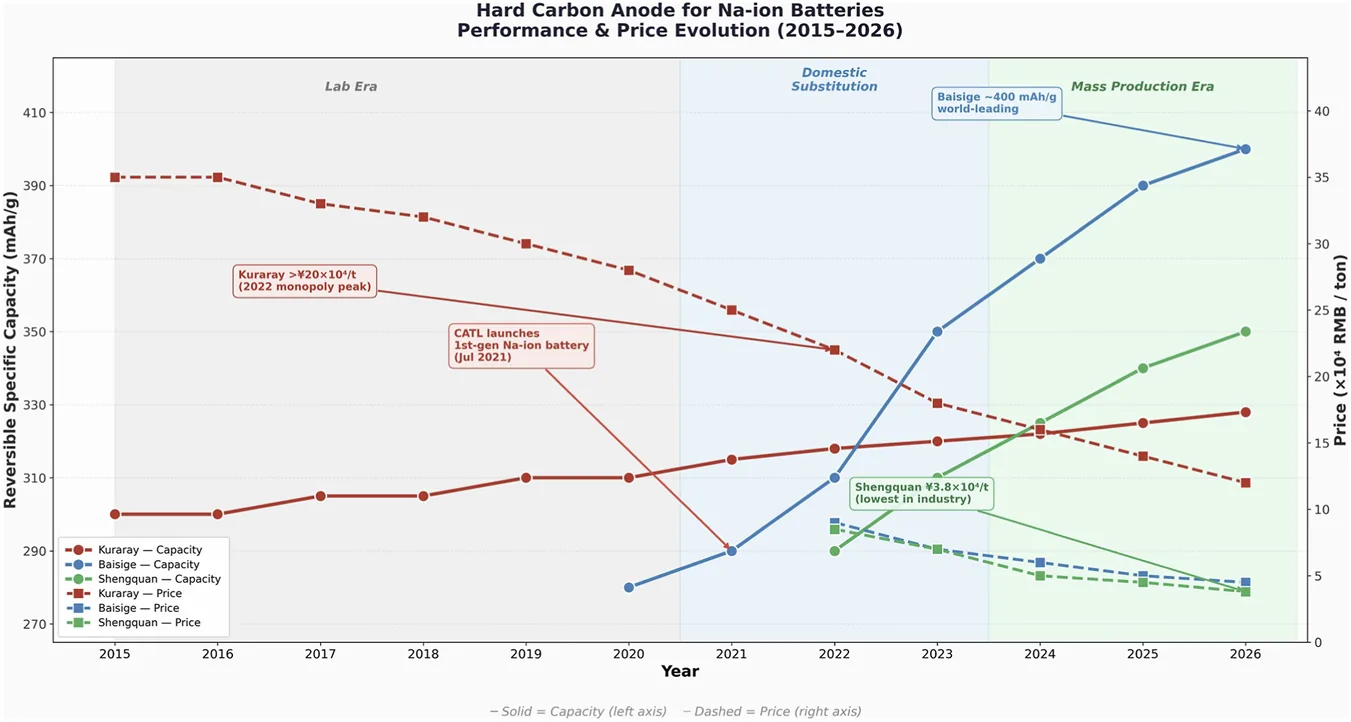
The price performance trend chart of some head hard carbon use companies in the past 10 years.

**TABLE 3:** Comparison of synthesis processes and electrochemical performance parameters of hard carbon anodes from major material manufacturers.

Company	Precursor	Synthesis method	Sp. Capacity (mAh g^-1^)	ICE
Chengdu baisige technology Co., Ltd	Starch, coconut shell, coal, resin, pitch	Acid pickling and deashing, pre-carbonization, ball milling, high-temp. Carbonization, pitch/resin liquid-phase coating, De-agglomeration and classification, electromagnetic iron removal	300–400	88%–92%
Sanquan group	Straw, cellulose	Pre-treatment (crushing/washing), Hydrothermal/LT pre-carbonization, high-temp. Carbonization, post-treatment crushing and classification	300–350	87%–90%
Shanshan co., ltd	Biomass	Precursor pre-treatment, pre-carbonization, high-temp. Carbonization, proprietary surface coating, 2nd carbonization pore-sealing, post-treatment	300–350	88%–90%
BTR new material group Co., Ltd	Coconut shell, pitch	Kureha technology transfer, in-house process adapt., pre-oxidation, carbonization and post-treat	300–350	87%–90%
Fujian yuanli active carbon Co. Ltd.	Coconut shell	Raw mat. Carbonization, activation/pore structure control	300–330	85%–88%
Shanghai Putailai new energy technology Co., Ltd	Hard carbon, soft carbon, biomass-derived graphite	CVD porous carbon framework (core tech. Mastered), carbonization/post-treat	-	-
Kuraray	Coconut shell	Proprietary carbonization, TYPE1 (9um)/TYPE2 (5/9um) precision classification, surface treatment (excellent moisture resistance)	300–330	88%–91%
Kureha	Petroleum-based, pitch	Pre-oxidation/crosslinking (prevents melting, high-temp. Carbonization, post-treatment	280–320	86%–89%
E-power resources	Plant-based	Multi-element doped HC composite, high-temp. Carbonization	-	-
Wuhan BYD	Phenolic resin	Low-temperature pre-firing, calcination at 600 °C–1500 °C	400–500	88%–90%
Jinan shengquan group Co., Ltd	Straw, phenolic resin	Resinification, calcination	400–500	90%–92%

In terms of supply structure, battery cell manufacturers’ single-year hard carbon demand will soon exceed the ten-thousand-ton level, so supply chain concentration will inevitably increase, and the number of core suppliers surviving will not exceed three to four. Kuraray and Wu Yu will retreat to high-end research channels in China, reducing their market share to single digits. If hard carbon startups in India and North America fail to complete production line validation before 2029, the window will be completely closed. By 2031, global hard carbon demand will be about 200,000–300,000 tons, with the Chinese market accounting for over 80% of production and sales, industry average prices of 30,000–35,000 yuan ton^−1^, and gross margins narrowing to 20%–25%. Hard carbon will transform from a “bottleneck” into a ten-billion-yuan basic materials track with a tentastic structure.

## 5 Conclusion and outlook

This review has traced the development of hard carbon anodes from sodium-storage mechanisms and precursor chemistry to microstructure regulation and industrial scale-up. The apparent contradictions among the insertion–filling, adsorption–insertion, and adsorption–filling models can be reconciled within a unified adsorption–intercalation–filling framework. Sodium storage involves the cooperative contribution of defect and surface adsorption, interlayer intercalation, and quasi-metallic sodium clustering in closed nanopores, with their relative contributions governed by the interlayer spacing, closed-pore volume, pore-wall defects, and carbonization history. At the same time, precursor chemistry provides the fundamental structural basis for hard-carbon formation. The distinct pyrolysis behaviors of cellulose, hemicellulose, and lignin, together with the controllable crosslinking characteristics of resin and pitch precursors, establish important links between precursor molecular structure, carbon microstructure, and electrochemical performance.

Despite the rapid progress in hard carbon research, its successful commercialization depends not only on further improvements in reversible capacity and initial Coulombic efficiency, but also on the establishment of scalable, stable, and economically viable manufacturing processes. Hard carbon production has already advanced from laboratory-scale synthesis toward industrial-scale manufacturing, with production capacity reaching the 10,000-ton level in some regions and China emerging as a major production center. In addition, the cost of hard carbon is approaching that of graphite, indicating increasingly favorable prospects for its application in sodium-ion batteries. Nevertheless, challenges remain in achieving consistent precursor quality, precise control of pore structure and surface chemistry, low energy consumption during carbonization, and reproducible electrochemical performance across large-scale batches. Future efforts should therefore shift from material screening toward integrated process engineering, including standardized precursor evaluation, continuous production, optimized carbonization and surface-treatment processes, and comprehensive techno-economic and life-cycle assessments. Through closer coordination among precursor suppliers, material manufacturers, battery producers, and end users, hard carbon is expected to develop into a reliable and commercially competitive anode material for large-scale sodium-ion battery applications.
